# Range of motion of resistance exercise affects the number of performed repetitions but not a time under tension

**DOI:** 10.1038/s41598-021-94338-7

**Published:** 2021-07-21

**Authors:** Michał Krzysztofik, Patryk Matykiewicz, Aleksandra Filip-Stachnik, Kinga Humińska-Lisowska, Agata Rzeszutko-Bełzowska, Michał Wilk

**Affiliations:** 1 Institute of Sport Sciences, The Jerzy Kukuczka Academy of Physical Education, ul. Mikolowska 72a, 40-065 Katowice, Poland; 2 Faculty of Physical Education, Gdansk University of Physical Education and Sport, Gdansk, Poland; 3 College of Medical Sciences, Institute of Physical Culture Studies, University of Rzeszow, Rzeszow, Poland

**Keywords:** Health occupations, Public health

## Abstract

The resistance training volume along with the exercise range of motion has a significant impact on the training outcomes. Therefore, this study aimed to examine differences in training volume assessed by a number of performed repetitions, time under tension, and load–displacement as well as peak barbell velocity between the cambered and standard barbell bench press training session. The participants performed 3 sets to muscular failure of bench press exercise with the cambered or standard barbell at 50% of one-repetition maximum (1RM). Eighteen healthy men volunteered for the study (age = 25 ± 2 years; body mass = 92.1 ± 9.9 kg; experience in resistance training 7.3 ± 2.1 years; standard and cambered barbell bench press 1RM > 120% body mass). The t-test indicated a significantly higher mean range of motion for the cambered barbell in comparison to the standard (*p* < 0.0001; ES =  −2.24). Moreover, there was a significantly greater number of performed repetitions during the standard barbell bench press than cambered barbell (*p* < 0.0001) in a whole training session, while no difference was found in total time under tension (*p* = 0.22) and total load–displacement (*p* = 0.913). The two-way repeated-measures ANOVA indicated a significant *barbell* × *set* interaction effect for peak velocity (*p* = 0.01) and a number of repetitions (*p* = 0.015). The post-hoc analysis showed a significantly higher number of repetitions for standard than cambered barbell bench press in set 1 (*p* < 0.0001), set 3 (*p* < 0.0001) but not in set 2 (*p* = 0.066). Moreover, there was a significantly higher peak velocity during the cambered than standard barbell bench press in set 1 (*p* < 0.0001), and set 2 (*p* = 0.049), but not in set 3 (*p* = 0.063). No significant differences between corresponding sets of the standard and cambered barbell bench press in time under tension and load–displacement were found. However, concentric time under tension was significantly higher during cambered barbell bench press in all sets (*p* < 0.05) when compared to the standard barbell bench press, while eccentric time under tension was significantly lower during the cambered than standard barbell bench presses only in the set 3 (*p* = 0.001). In summary, this study briefly showed that measuring training volume by the number of performed repetitions is not reliable when different exercise range of motion is used.

## Introduction

Resistance training volume is a variable that plays a significant role in inducing muscle adaptations such as hypertrophy as well as strength^1^. Nevertheless, quantifying training volume is an issue for the fitness community. Training volume can be determined in several ways, based on the a) total work, b) the number of performed repetitions (REP), c) the tonnage or d) the time under tension. It seems that the total work (force [N] × displacement [m]) is the most accurate, however, it requires the use of expensive devices thus in a real-life scenario almost impossible. An easier solution is tonnage, which is calculated by multiplying the number of performed REPs by the workload in kilograms. An alternate method used is just counting the number of REPs performed. However, in both cases, the duration of a single REP and load–displacement is not taken into account. The duration of performed REP is not always the same, since an increase in load or range of motion (ROM), as well as an increase in fatigue, may cause an extension in the duration of the movement^2,3^. Therefore, the tempo in particular phases of the movement should be fixed. A study by Wilk et al.^4^ showed significant differences in training volume measured as the number of REPs as well as time under tension when different movement tempos were used during a bench press. Briefly, that the higher number of REPs does not always translate into a longer time under tension. Furthermore, it also means that an equal number of REPs does not have to correspond to the same amount of time under tension^5^. Moreover, the influence of exercise ROM on the duration of REP seems logical and it was confirmed in the literature^6^. A study by Krzysztofik et al.^6^ showed that, despite the equal number of performed REPs, a significantly longer ROM caused by the use of a cambered barbell during bench press exercises, resulted in a significantly greater time under tension in comparison to standard barbell bench press. However, participants in this study performed low-volume bench press (a single set of 3 repetitions), so it cannot be ruled out that differences might arise if higher exercise volumes would be used. Therefore, bearing in mind the above-mentioned aspects and according to Wilk et al.^4,5^, time under tension might be the most reliable indicator to assess exercise volume in resistance exercise regardless of the number of performed REPs and desired ROM.

The effects of the ROM on training outcomes have been widely analyzed^7–13^. A study by Martínez-Cava et al.^11,12^ indicated that the mean velocity achieved against a wide range of loads was significantly higher, with a greater ROM during resistance exercises. In regards to the long-term adaptations, Pallarés et al.^13^ found that 10 weeks of full ROM back squat exercise produced greater improvements in jump height, as compared to partial ROM in that exercise. Similarly, a study by Martínez-Cava et al.^11^ showed that 10 weeks of full ROM bench press exercises led to greater improvements in maximum strength compared to the partial ROM bench press. However, experimental procedures of mentioned studies equalized training volume to the number of performed REPs, which means that when performing an exercise with a larger ROM, more work was done. Thus, the findings may not be the result of the ROM itself, but a larger training volume performed in full ROM conditions. While when training volume was not equalized to the number of REPs between conditions, the findings from studies comparing different ROMs are inconsistent^8,10^. For example, Kubo and colleagues^10^ compared the effectiveness of a 10-week full and partial back squats training on lower limb muscle development with equalized training volume between conditions to load × repetition × displacement instead of REPs only. Admittedly, as in previous studies, authors also found a greater increase in muscle strength and hypertrophy in full than partial ROM back squat training. In turn, Valamatos et al.^8^ investigated the effect of a 15-week seated knee extensions training performed with full or partial ROM with training volume equalized to the time under tension. The results showed a significant difference in muscle architectural parameters but no significant differences were found in muscle volume and strength.

Interestingly, in terms of ROM, the aspect of the limitations caused by training equipment has not been raised. While in the case of the barbell squats, the physiological capabilities of a joint or several joints limit the achievement of a large squat depth, in bench press it is a barbell. The ROM is restricted by the barbell which touches the chest and for this reason, it does not allow to reach the “real” full ROM. Concerning that, the major muscles engaged in the bench press exercise are not going through their full physiological ROM. To overcome this limitation, a cambered barbell has been designed. The cambered barbell is U-shaped, which provides additional space for the torso allowing it to reach a lower bottom position in comparison to the standard barbell^6,14^, thus achieving greater stretch of the chest and shoulders muscles. Even though the cambered barbell has been around for a long time, and the bench press exercise is one of the most studied and commonly used upper-body exercises in training^15^, so far there has been little research into its use in training^6,16^. To date, studies analyzed changes in muscle activity during cambered barbell and standard barbell bench press or assess the differences in power output and barbell velocity. A study by Krzysztofik et al.^16^ found that during the cambered barbell bench press, the anterior deltoid is activated to a greater extent than during the standard barbell bench press, while the standard barbell contributes to the greater pectoralis major and triceps brachii long head muscle activity. In addition, recent studies have shown that the use of a cambered barbell during bench press allows achieving significantly higher barbell velocity values compared to standard barbell^6^. However, to date, no studies have assessed the difference in training volume achieved between the cambered barbell and standard barbell bench press.

Considering the common use of bench press exercise in means of upper-body muscular development and that the cambered barbell can significantly affect the muscle activity and barbell velocity during this exercise due to differences in ROM^6,16^, the purpose of this study was to assess the impact of the cambered barbell during the bench press on the training volume based on the total number of performed REPs, time under tension, and load–displacement. We hypothesized that a cambered barbell bench press would have a significant effect on a number of performed REPs but not on a time under tension and load–displacement (load × repetition × displacement) for individual sets and the entire training session.

### Participants

Eighteen healthy well-trained in resistance training men participated in this study (Table 1). The inclusion criteria were: i) a bench press personal record of at least 120% of body mass ii) participation in resistance training at least 3 days per week for the 6 months immediately before enrollment in this study iii) at least 3 weeks of previous experience with a cambered barbell bench press (to avoid of the learning effect of the bench press exercise technique^17^). The study participants were allowed to withdraw from the experiment at any moment and were free from musculoskeletal disorders. They were informed about the benefits and potential risks of the study before providing their written informed consent for participation. The study protocol was approved by the Bioethics Committee for Scientific Research, at the Academy of Physical Education in Katowice, Poland (10/2018), and performed according to the ethical standards of the Declaration of Helsinki, 2013. To calculate the sample size, statistical software (G*Power, Dusseldorf, Germany) was used. Given the study 2-way analysis of variance (ANOVA) (2 condition and 3 repeated measures), a small overall effect size (ES) = 0.35, an alpha-error < 0.05, and the desired power (1-ß error) = 0.8, the total sample size resulted in 16 participants.

**Table 1:** Descriptive characteristics of the study participants.

Age (years)	25 ± 2
Body mass (kg)	92.1 ± 9.9
Experience in RT (years)	7.3 ± 2.1
Standard Barbell 1RM (kg)	140 ± 17
Standard Barbell ROM (cm)	35 ± 2.3
Cambered Barbell 1RM (kg)	133 ± 16
Cambered Barbell ROM (cm)	41 ± 2.9

*RT* resistance training, *1RM* one repetition maximum, *ROM* range of motion.

### Study design and procedure

**Figure 1:**
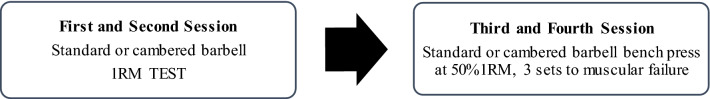
Schematic representation of the experimental sessions protocol.

**Figure 2:**
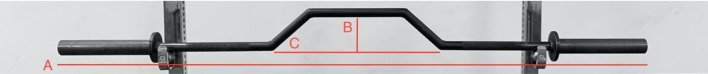
The cambered barbell characteristics as previously presented elsewhere^6^. Weight—20 kg; (**A**) overall length—190 cm; (**B**) camber depth—10 cm; (**C**) space between camber—55 cm.

All of the participants were familiar with the cambered barbell bench press exercise since they had participated in previous cambered barbell studies or performed a cambered barbell bench press in their regular workout routine. The participants took part in four experimental sessions within 3 weeks. The first and second sessions included the determination of the one-repetition maximum (1RM) load of the flat bench press with the standard barbell or cambered barbell, 96 h apart. After the next 96 h, the third session began and the fourth after an additional week (from the third session). These sessions consisted of performing the 3 sets of bench press exercise with the cambered or standard barbell at 50%1RM to muscular failure (Fig. 1). During each set, the number of performed repetitions, time under tension, load–displacement, and peak velocity were recorded. The participants were instructed not to perform any additional resistance exercises within 72 h of testing to avoid fatigue. Moreover, they were asked to maintain their normal dietary and sleep habits throughout the study and not to use any supplements or stimulants for 24 h prior to the sessions. The cambered barbell features are presented in the Fig. 2.

### Experimental sessions

Two 1RM testing sessions were used for the experimental trials and the protocols were identical except for the use of standard barbell or cambered barbell during bench press exercise. All testing trials were conducted at the same time of the day to avoid circadian variation (in the morning between 9:00 and 11:00 am) and were separated by a 96 h interval. The standardized warm-up was performed before each experimental session as described elsewhere^18^. After that, the participants performed the 1RM bench press test with standard or cambered barbell to assess upper-body maximal muscle strength. During that, the participants executed a single repetition with a constant tempo of movement (2 s duration of eccentric phase and maximal speed in the concentric phase, with no pause in-between) and standardized hand placement on the barbell (150% individual bi-acromial distance). The loading started at 80% estimated 1RM and if the participant successfully lifted the load, the weight was increased by 2.5 to 10 kg in subsequent attempts until the 1RM for a particular session was obtained. The 1RM was defined as the highest load completed without any help of the spotters^19–21^. Five-minute rest intervals were used between the 1RM attempts, and all 1RM values were obtained within five attempts.

During the third and fourth sessions, the participants completed 3 sets of bench press exercises with either standard or cambered barbell to momentary muscular failure with a load equivalent to 50% of the participants’ 1RM, as measured previously in the 1RM test. The rest interval between sets equaled 5 min. Muscular failure was defined as the inability to perform another concentric movement in its entire range of motion^22^. To ensure safety and technical proficiency, the two strength and conditioning specialists were present during all attempts. The eccentric phase of each repetition was performed with a constant duration of 2 s, while the concentric phase at maximal possible velocity, but without bouncing the barbell off the chest, without intentionally pausing at the transition between the eccentric and concentric phases^19,23^. It should be emphasized that since not all participants were able to touch their chests during the cambered barbell bench press, they were instructed to lower the barbell to a range that was comfortable for them. In considering this issue and participants safety, the eccentric phase was standardized to 2 s.

A linear position transducer system (Tendo Power Analyzer, Tendo Sport Machines, Trencin, Slovakia) was used for the evaluation of barbell velocity and ROM during the bench press exercise. The system consists of a velocity sensor connected to the barbell with a kevlar cable, which, through the interface, immediately transmits the vertical velocity reached by the barbell to software installed on the computer. The sampling rate is determined by the velocity of the disk’s rotation (for example 200 Hz for 2 m/s). In previous studies, this linear transducer has emerged as a reliable system for measuring barbell velocity and power output during bench press exercises (intra-class correlation coefficient ∼ 0.905–0.989)^24,25^. The peak barbell velocity was obtained as the average of peak velocity from all repetitions performed in particular sets, while the ROM was reported as the mean of all repetitions performed in a whole session. Time under tension was obtained manually from the data recorded by a camera (Sony FDR191 AX53), using slow-speed playback (1/5 speed). In order to ensure the reliability of manual data collection, three independent persons made the data analysis from the camera.

### Statistical analysis

All calculations were performed using SPSS (version 25.0; SPSS, Inc., Chicago, IL, USA) and were expressed as means with standard deviations (± SD). Statistical significance was set at *p* < 0.05. The paired samples t-test was performed to assess differences in the mean ROM, 1RM, total number of performed REPs, total time under tension, total concentric as well as eccentric time under tension, and load–displacement between the cambered and standard barbell bench press to muscular failure. The normality of data distribution was checked using Shapiro–Wilk tests. Due to the normal distribution of all analyzed data, the number of performed repetitions, time under tension, and peak velocity were analyzed with a two-way (*barbell* × *set*; 2 × 3) ANOVA with repeated measures. In the event of a significant main effect, post-hoc comparisons were conducted using the Bonferroni test. The magnitude of mean differences was expressed with standardized (Hedges g) effect sizes; thresholds for qualitive descriptors of Hedges’s *g* were defined as ≤ 0.20 as “small”, 0.21–0.8 “medium”, and > 0.80 as “large”. Mauchly’s test of sphericity was conducted to test for the homogeneity of data and if violated (p < 0.05), the Greenhouse–Geisser adjustment value was used. The 95% confidence intervals for mean values were also calculated.

## Results

Table 2 contains the differences in performance variables during the cambered and standard barbell bench press exercise. The t-test indicated significantly higher mean ROM for the cambered barbell in comparison to the standard (41 ± 2.9 cm vs. 35 ± 2.3; *p* < 0.0001; ES =  −2.24). Furthermore, a significantly higher 1RM value was obtained during the standard than the cambered barbell bench press (140 ± 17 vs. 133 ± 16; *p* < 0.0001; ES =  −0.41). Moreover, there was a significantly greater number of performed REPs during the standard barbell bench press than cambered (*p* < 0.0001) in a whole training session, while no difference was found in total time under tension (*p* = 0.22) and total load–displacement (*p* = 0.913) (Table 2). However, the total concentric time under tension was significantly higher during the cambered barbell bench press than standard (*p* < 0.0001), while the total eccentric time under tension was higher in the standard barbell bench press than cambered (*p* = 0.01) in a whole training session (Table 2). The two-way repeated measures ANOVA indicated a significant *barbell* × *set* interaction effect for peak velocity (*p* = 0.01) and a number of repetitions (*p* = 0.015). Overall, there was a main effect of the set to decrease the number of repetitions (*p* < 0.0001), time under tension (*p* < 0.0001), concentric time under tension (*p* < 0.0001), eccentric time under tension (*p* < 0.0001), load–displacement (*p* < 0.0001), and peak velocity (*p* < 0.0001) across the workout. The post-hoc analysis showed a significantly higher number of repetitions for standard than cambered barbell bench press in set 1 (*p* < 0.0001), set 3 (*p* < 0.0001) but not in set 2 (*p* = 0.066) (Table 3). No significant differences between corresponding sets of the standard and cambered barbell bench press in time under tension and load–displacement were found. However, concentric time under tension was significantly higher during cambered barbell bench press in all sets (*p* < 0.05) when compared to the standard barbell bench press, while eccentric time under tension was significantly lower during the cambered than standard barbell bench press only in the set 3 (*p* = 0.001). Moreover, there was a significantly higher peak velocity during the cambered than standard barbell bench press in set 1 (*p* < 0.0001), and set 2 (*p* = 0.049), but not in set 3 (*p* = 0.063).

**Table 2:** Differences in whole training session volume variables measured during 3 sets of the cambered barbell and standard barbell bench press.

Standard Barbell (95%CI)	Cambered Barbell (95%CI)	Significance
Total number of repetitions (n)		
59.1 ± 5 (56.6–61.6)	53.1 ± 5.4* (50.4–55.8)	0.0001
Total time under tension (s)		
156.6 ± 14.9 (149.2–164)	159.5 ± 17 (150.9–167.9)	0.22
Total concentric time under tension (s)		
45.3 ± 8.1 (41.2–49.3)	54.8 ± 13* (48.4–61.3)	< 0.0001
Total eccentric time under tension (s)		
111.3 ± 9.4 (106.7–116)	104.7 ± 10.6* (99.1–109.6)	0.01
Total load displacement (kg × cm)		
144318 ± 17789 (135471–153164)	144814 ± 20167 (134785–154843)	0.913

*CI* confidence interval.

*p < 0.05 significant different from standard barbell bench press.

**Table 3:** Differences in performance variables during a standard and cambered barbell bench press.

	Standard Barbell (95%CI)	Cambered Barbell (95%CI)	ES	Barbell	Set	Interaction
Repetition (n)						
Set 1	25.8 ± 2.5 (24.6–27.07)	23.3 ± 1.9* (22.4–24.3)	− 1.1	< 0.0001	< 0.0001	0.015
Set 2	17.7 ± 1.7 (16.8–18.5)	16.7 ± 2.2 (15.6–17.8)	− 0.5			
Set 3	15.6 ± 1.7 (14.7–16.4)	13.2 ± 1.9* (12.2–14.1)	− 1.3			
Time under tension (s)						
Set 1	65.9 ± 8.1 (61.9–69.9)	66.5 ± 6.4 (63.3–69.7)	0.08	0.22	< 0.0001	0.797
Set 2	48.6 ± 4.5 (46.3–50.9)	50.3 ± 6.1 (47.2–53.3)	0.31			
Set 3	42.1 ± 4.6 (39.8–44.4)	42.7 ± 6.8 (39.3–46)	0.1			
Concentric time under tension (s)						
Set 1	17.4 ± 4.3 (15.3–19.5)	19.7 ± 4.8* (17.3–22)	0.49	< 0.0001	< 0.0001	0.212
Set 2	14.6 ± 2.4 (13.5–15.8)	17.6 ± 3.7* (15.8–19.5)	0.94			
Set 3	13.3 ± 3.1 (11.6–14.7)	17.5 ± 6.1* (14.5–20.5)	0.85			
Eccentric time under tension (s)						
Set 1	48.5 ± 5.4 (45.8–51.2)	46.8 ± 4.9 (44.4–49.3)	− 0.32	0.01	< 0.0001	0.276
Set 2	34 ± 3.5 (32.3–35.7)	32.6 ± 3.9 (30.4–34.3)	− 0.37			
Set 3	28.8 ± 2.7 (27.5–30.2)	25.3 ± 3.9* (23.2–27.1)	− 1.02			
Peak velocity (m/s)						
Set 1	0.69 ± 0.06 (0.66–0.73)	0.76 ± 0.06* (0.73–0.79)	1.14	0.004	< 0.0001	0.01
Set 2	0.67 ± 0.05 (0.64–0.69)	0.7 ± 0.06* (0.66–0.73)	0.53			
Set 3	0.64 ± 0.06 (0.61–0.67)	0.68 ± 0.07 (0.64–0.71)	0.6			
Load displacement (kg × cm)						
Set 1	63851 ± 10082 (58838–68865)	64015 ± 10961 (58564–69466)	0.02	0.913	< 0.0001	0.176
Set 2	43278 ± 5471 (40558–45999)	45250 ± 6744 (41897–48604)	0.31			
Set 3	37188 ± 4405 (34997–39379)	35548 ± 4596 (33263–37834)	− 0.36			

*CI* confidence interval, *ES* effect size.

*p < 0.05 significant different from the corresponding value in the standard barbell bench press.

## Discussion

The main finding of this study was that a significantly higher number of performed REPs was achieved during standard than the cambered barbell bench press, however, there were no significant differences in time under tension and load–displacement. Since the REP may lead to an inaccurate assessment of training volume due to the omission of the differences in ROM, it seems that time under tension is a more accurate indicator of training volume in non-laboratory situations and should be used to equalize the volume in studies assessing the effect of ROM on training outcomes. In addition, a significantly higher peak velocity in the first and second set during the cambered barbell bench press was demonstrated than in the standard barbell. Furthermore, the distribution of concentric and eccentric time under tension differed between the cambered and standard barbell bench press.

These results provide valuable insight for trainers and practitioners on training volume, which is the variable that plays a major role in training adaptations^26,27^. Drawing conclusions from this study based on the number of performed REPs, with omitting the greater ROM due to the use of cambered barbell during bench press, one could conclude that this barbell contributes to obtaining a significantly lower training volume. However, the time under tension and load–displacement values obtained contradict this statement and indicate that there were no significant differences in training volume performed. Therefore, comparing the training volume between similar exercises performed with different ROMs (i.e., full vs. partial bench press) or equating it solely based on the number of REPs performed, to compare the effectiveness of those exercises on training outcomes may provide misleading findings. Although previous studies have already confirmed that time under tension is a more accurate measure of training volume than the number of performed REPs, they focused mainly on the assessment of the impact of movement tempo on the differences in training volume, both in terms of the number of performed REPs and the duration of time under tension^4,5,28^. However, data on the impact of the ROM on the achieved training volume are missing. These findings should be taken into account especially when designing research procedures comparing the effectiveness of different ROM in a given exercise on the training outcomes. The results of this type of studies rather indicated a superior effect of the full ROM compared to the partial one on muscle development^10–13,29^, with some exceptions^8,9^. However, only one of these studies equalized the training volume to the time under tension^8^ or load–displacement^10^ while the vast majority did so based on the number of performed REPs^9,11–13^. In our study, it was shown that the use of a cambered barbell during bench press extended the ROM by an average of 6 cm, meaning that the participant had to cover a 12 cm longer displacement in each repetition. In the case of a typical training session consisting of e.g., 4 sets of 8 repetitions, this gives a longer total displacement of 384 cm, which corresponds to an additional set. Causing that a greater training volume would be performed throughout the training session, and indeed the differences in the training outcomes would not be due to the influence of the ROM, but to the training volume. Therefore, it seems that in a non-laboratory situation, when an assessment of total work is impossible (force × displacement) more reliable is to use time under tension as a measure of training volume than a number of performed REPs. Nevertheless, the number of performed REPs can still be considered as a good indicator of the training volume, but only when the conditions of the exercise are the same, i.e., the ROM and the tempo movement of each phase of the exercise is established.

Also, interesting and somewhat surprising, is that the ROM of the performed exercise has no effect on when a muscular failure occurs. It would seem that the higher the number of performed REPs during the standard barbell bench press, and thus the more frequent occurrence of the stretch–shortening cycle, will contribute to achieving a longer time under tension and load–displacement. However, no significant differences in these variables were found between standard and cambered barbell bench press. Since more frequent use of the stretching and shortening cycle allows more energy to be stored and released from elastic components such as tendons and ligaments, and thus lower metabolic costs requirements^30,31^, it could be speculated that muscular failure occurs earlier during cambered than a standard barbell bench press, but it wasn’t. Nevertheless, it should be highlighted that the analysis showed a significant difference in the distribution of time under tension in concentric and eccentric phases between the cambered and standard barbell bench press. Therefore, it cannot be excluded those differences would arise if the experimental protocol assumed a larger number of sets than used in this study (> 3 sets).

Should also be mentioned that the significantly higher velocity of the barbell was obtained in the first and second set of cambered barbell bench press than in the standard barbell. This is in line with the research carried out so far^6^. A study by Krzysztofik et al.^6^ showed that the cambered barbell significantly increased barbell velocity in the bench press exercise at 50%1RM compared to the standard barbell. This is due to the additional ROM that allows the barbell to be accelerated by a considerably greater displacement, which has a positive effect on the achieved velocity. However, it is interesting why such changes were not reported in set 3. Since, both the cambered and standard barbell bench press, was performed till muscular failure, it may have been assumed that the differences in barbell velocity will be maintained in particular sets. This is probably because, in the case of a cambered barbell there is an extended eccentric phase for the major muscles engaged during bench press (pectoralis major, triceps brachii, anterior deltoid), which can lead to greater muscle damage^32^. Moreover, it should be highlighted that a significantly lower value of 1RM was obtained during the cambered than the standard barbell bench press. This is in line with previous research showing that the greater the ROM, the lower the 1RM value^11,13^. Moreover, the presence of the sticking region is also affected by ROMs, and larger ROMs may make them longer^33^. Thus, it seems that different ROMs resulted in a longer sticking region during the cambered in comparison to the standard barbell bench press, which may underlie this disadvantage.

Our study is not without limitations. First of all, we have compared only two different ROMs in the bench press, and only a single value of external load was used. Therefore, the results of the presented study may not translate to other loads or exercises. Moreover, we have not analyzed power- and velocity-load relationships as well as biomechanical factors which may affect performance during the cambered barbell bench press. What's also important, we didn't compare these results with the training volume counted as total work (force × displacement). Future research could compare the impact of 3 different ROMs on training volume, extended (by using a cambered barbell), full in terms of a standard barbell, and partial (by using an inverse cambered barbell).

In summary, this study briefly showed that measuring volume by the number of performed REPs is not reliable when different ROM of exercises is used. Therefore, in order to properly control the training volume in such cases, it is recommended to use the time under tension. The results of our research and previous scientific reports indicate that the duration of time under tension is a reliable method of measuring training volume, which should be taken into account when planning and following training routines. We suggest that similar protocols should be replicated with other exercises such as full and partial back squats to verify these findings.
